# 

**Published:** 1871-10

**Authors:** 


### Books and Pamphlets Received.

Practical Therapeutics: considered chiefly with reference to articles of the Materia Medica. By Edward John Waring, M.D., F.L.S. Second American from the third London edition.—Philadelphia, Lindsay & Blakiston, 1871. Buffalo: Breed, Lent & Co.

The Management of Infancy, Physiological and Moral; intended chiefly for the use of parents. By Andrew Combe, M.D. Revised and edited by Sir James Clark, Bart., K.C.B., M.D., F.R.S. First American from the Tenth London edition. New York: D. Appleton & Co., 1871. Buffalo: Breed, Lent & Co.

Skin Diseases; their description, pathology, Diagnosis and treatment. By Tilbury Fox, M.D., London, M.R.C.P. First American from the last London edition. Edited by M. H. Henry, M.D. New York: William Wood & Co., 1871 Buffalo: Breed, Lent & Co.

Essentials of the Principles and Practice of Medicine. A hand-book for the students and practitioners. By Henry Hartshorne, A.M., M.D. Third edition thoroughly revised. Philadelphia: Henry C. Lea, 1871. Buffalo: Breed, Lent & Co.

Pocket Anatomist: containing a concise description of the structure of the human body. Third edition with corrections and additions. By C. E. Isaacs, M.D. New York: William Wood & Co., 1871. Buffalo: Breed, Lent & Co.

Medical Education in America; being the Annual Address read before the Massachusetts Medical Society, June 7th, 1871. By Henry J. Bigelow, M.D. Cambridge: Welch, Bigelow & Co., 1871.

Thoughts on Chronic Inversion of the Uterus. By Henry Miller, M.D. Louisville, Ky.

Electrolysis, and its application to the treatment of disease. By A. D. Rockwell, A.M., M.D. New York: D. Appleton & Co.

Introductory Lecture to the course on Pathological Anatomy at the University of Pennsylvania. By Joseph G. Richardson, M.D. Philadelphia: J. B. Lippincott & Co.

Prolapsus Uteri, its chief causes and treatment. By Thomas Addis Emmett, M.D.

Albany Hospital. A statement from the Governors of its History and present condition.

Contributions to Practical Laryngoscopy. By A. Ruppaner, A.M., M.D.

PUBLISHED MONTHLY,

Containing Original Articles, Reports of Medical Societies and Hospitals, Editorial, Reviews, Correspondence, Army News, etc., etc ; including the usual variety of Medical Periodical Publications. Specimen copies sent on application.

Address, Buffalo Medical & Surgical Journal, Buffalo, N. Y.

TERMS OF SUBSCRIPTION, - - $3.00 PER YEAR, IN ADVANCE.

